# Supplementary material for: Prediction of amphipathic helix—membrane interactions with Rosetta
Source: PLoS Comput Biol. 2021 Mar 17;17(3):e1008818. doi: 10.1371/journal.pcbi.1008818 (PMC8007005; doi:10.1371/journal.pcbi.1008818)
Supplement: S1 Table — The “_xn” denotation was used to distinguish multiple helices belonging to the same protein structure. (DOCX) [file pcbi.1008818.s001.docx]

## Supporting Tables

Supporting Table 1: List of the amphipathic helices in the benchmark set and their corresponding residue numbers and chain IDs in the PDB files. The “_xn” denotation was used to distinguish multiple helices belonging to the same protein structure.

| Helix name | PDB region |
| --- | --- |
| 1b4v_h1 | 78-87.A |
| 1h0a_h1 | 1-18.A |
| 1q4g_h1 | 73-83.A |
| 1q4g_h2 | 85-95.A |
| 1q4g_h3 | 96-106.A |
| 1q4g_h4 | 107-123.A |
| 1rhz_h1 | 2-23.B |
| 2hih_h1 | 190-206.A |
| 2ziy_h1 | 320-329.A |
| 3a7k_h1 | 20-29.A |
| 3hyw_h1 | 380-395.A |
| 3hyw_h2 | 400-412.A |
| 3i9v_h1 | 18.6-33.6 |
| 3j5p_h1 | 559-575.A |
| 3jw8_h1 | 167-181.A |
| 3tij_h1 | 50-78.A |
| 4hhr_h1 | 1-15.A |
| 4hhr_h2 | 9-44.A |
| 4hhr_h3 | 51-70.A |
| 4m5e_h1 | 160-172.A |
| 4nwz_h1 | 369-388.A |
| 4qnd_h1 | 1-13.A |
| 4rp9_h3 | 252-261.A |
| 4umw_h1 | 165-176.A |
| 4ymk_h1 | 128-141.A |
| 4ymk_h2 | 281-288.A |
| 4ymk_h3 | 315-326.A |
| 4zwn_h1 | 156-176.A |
| 5ahv_h1 | 1-16.E |
| 5dqq_h1 | 198-213.A |
| 5ek8_h1 | 1-23.A |
| 5f19_h3 | 96-105.A |
| 5f19_h4 | 106-122.A |
| 5lil_h1 | 246-263.A |
| 5mlz_h2 | 213-229.A |
| 5uz7_h1 | 395-418.R |
| 5w7b_h1 | 159-179.C |
| 5w7l_h1 | 82-91.A |
| 5w7l_h2 | 93-104.A |
| 5w7l_h3 | 166-179.A |
| 6an7_h1 | 3-19.D |
| 6d26_h1 | 2309-2325.A |
| 6dvy_h1 | 681-708.A |
| 6igk_h1 | 302-403.A |
