# Supplementary material for: Prediction of amphipathic helix—membrane interactions with Rosetta
Source: PLoS Comput Biol. 2021 Mar 17;17(3):e1008818. doi: 10.1371/journal.pcbi.1008818 (PMC8007005; doi:10.1371/journal.pcbi.1008818)
Supplement: S2 Table — Positive depths correspond to helices inside the membrane and negative values indicate helices outside the membrane region. All units are in Angstroms (Å). (DOCX) [file pcbi.1008818.s002.docx]

Supporting Table 2: The depths calculated for the LK peptides using the *RosettaMembrane*, *ref2015_memb*, and *franklin2019* score functions. Positive depths correspond to helices inside the membrane and negative values indicate helices outside the membrane region. All units are in Angstroms (Å).

| Peptide | RosettaMembrane | ref2015_memb | franklin2019 |
| --- | --- | --- | --- |
| LK15 | 3.4 | 6.9 | 3.1 |
| LK18 | 4.1 | 6.2 | 4.1 |
| LK19 | 3.9 | 7.1 | 3.8 |
| LK20 | 3.8 | 5.1 | 4.2 |
| LK21 | 4.0 | 4.6 | 4.2 |
| LK22 | 3.0 | 6.6 | 3.6 |
