# Supplementary material for: Prediction of amphipathic helix—membrane interactions with Rosetta
Source: PLoS Comput Biol. 2021 Mar 17;17(3):e1008818. doi: 10.1371/journal.pcbi.1008818 (PMC8007005; doi:10.1371/journal.pcbi.1008818)
Supplement: S3 Table — The tilt angles for the first four peptides were taken from Ulmschneider et al. 2006. The tilt angle for WALP23 was taken from Ozdirekcan et al. 2005. The experimental tilt angles were converted to angle with the membrane plane for consistency. For the helices that had multiple energy minima, the lowest-scoring two were reported as the Tilt angle 1 and the Tilt angle 2. All angles are reported in degrees and all depths are reported in Ångstroms (Å). (DOCX) [file pcbi.1008818.s003.docx]

Supporting Table 3: The tilt angles and depths calculated for 1A11, 1PJD, 1PJE, 2NR1, and WALP23. The tilt angles for the first four peptides were taken from Ulmschneider et al. 2006. The tilt angle for WALP23 was taken from Ozdirekcan et al. 2005. The experimental tilt angles were converted to angle with the membrane plane for consistency. For the helices that had multiple energy minima, the lowest-scoring two were reported as the Tilt angle 1 and the Tilt angle 2. All angles are reported in degrees and all depths are reported in Ångstroms (Å).

| Helix | Experimental tilt angle | Rosetta Membrane Tilt Angle 1 | Rosetta Membrane Tilt Angle 2 | ref2015_memb Tilt Angle 1 | ref2015_memb Tilt Angle 2 | franklin2019 Tilt Angle 1 | franklin2019 Tilt Angle 2 |
| --- | --- | --- | --- | --- | --- | --- | --- |
| 1A11 | 78 | 8 | 90 | 88 | 30 | 86 | N/A |
| 1PJD | 86 | 72 | N/A | 70 | N/A | 54 | N/A |
| 1PJE | 74 | 42 | N/A | 60 | N/A | 14 | 68 |
| 2NR1 | - | 0 | 90 | 66 | 41 | 64 | N/A |
| WALP23 | 82 | 90 | N/A | 34 | 0 | 67 | N/A |
