# Supplementary material for: Prediction of amphipathic helix—membrane interactions with Rosetta
Source: PLoS Comput Biol. 2021 Mar 17;17(3):e1008818. doi: 10.1371/journal.pcbi.1008818 (PMC8007005; doi:10.1371/journal.pcbi.1008818)
Supplement: S4 Table — Positive depths correspond to helices below the membrane surface and negative values indicate helices outside the membrane region. All units are in Angstroms (Å). (DOCX) [file pcbi.1008818.s004.docx]

Supporting Table 4: The PDB IDs, helix ranges, and the calculated membrane depths of the tested hydrophilic peptides. Positive depths correspond to helices below the membrane surface and negative values indicate helices outside the membrane region. All units are in Angstroms (Å).

| PDB ID | Helix Range | RosettaMembrane Depth | Ref2015_memb Depth | Franklin2019 Depth |
| --- | --- | --- | --- | --- |
| 1FVN | 17-28 | -3.9 | -3.9 | 2.1 |
| 2B4N | 2-28 | -3.8 | -6.8 | 4.3 |
| 2KHK | 11-42 | -10 | -6.2 | -10 |
| 2MVM | 168-186 | -10 | -5.6 | -10 |
| 2O8Z | 30-41 | -5.8 | -3.6 | -10 |
| 5XNG | 1-17 | -2.6 | -2.9 | 0.3 |
