# Supplementary material for: Prediction of amphipathic helix—membrane interactions with Rosetta
Source: PLoS Comput Biol. 2021 Mar 17;17(3):e1008818. doi: 10.1371/journal.pcbi.1008818 (PMC8007005; doi:10.1371/journal.pcbi.1008818)
Supplement: S5 Table — (DOCX) [file pcbi.1008818.s005.docx]

Supporting Table 5: The RMSD values calculated for the best Rosetta zα- and zαβ-scan poses with OPM membrane thicknesses using *RosettaMembrane*, *ref2015_memb*, and *franklin2019* score functions.

| Name | RosettaMembrane RMSD zα | ref2015_memb RMSD zα | franklin2019 RMSD zα | RosettaMembrane RMSD zαβ | ref2015_memb RMSD zαβ | franklin2019 RMSD zαβ |
| --- | --- | --- | --- | --- | --- | --- |
| 1b4v_h1 | 2.1 | 12.0 | 7.1 | 3.9 | 12.0 | 7.2 |
| 1h0a_h1 | 0.5 | 1.7 | 1.7 | 2.2 | 2.1 | 1.6 |
| 1q4g_h1 | 3.6 | 4.8 | 1.2 | 3.6 | 4.8 | 1.4 |
| 1q4g_h2 | 3.2 | 2.7 | 8.3 | 3.7 | 2.2 | 8.8 |
| 1q4g_h3 | 4.2 | 8.0 | 1.8 | 4.2 | 10.2 | 3.5 |
| 1q4g_h4 | 13.3 | 10.3 | 16.1 | 5.7 | 12.1 | 17.0 |
| 1rhz_h1 | 2.8 | 1.7 | 4.7 | 2.2 | 2.2 | 9.4 |
| 2hih_h1 | 1.9 | 2.6 | 6.3 | 1.8 | 2.1 | 6.1 |
| 2ziy_h1 | 2.8 | 3.0 | 2.4 | 2.7 | 2.9 | 6.2 |
| 3a7k_h1 | 1.4 | 2.3 | 4.4 | 1.7 | 1.4 | 6.7 |
| 3hyw_h1 | 3.8 | 3.5 | 8.3 | 5.6 | 3.5 | 14.1 |
| 3hyw_h2 | 2.3 | 5.1 | 5.2 | 2.3 | 4.9 | 5.3 |
| 3i9v_h1 | 1.1 | 2.0 | 4.7 | 2.3 | 1.5 | 6.3 |
| 3j5p_h1 | 1.0 | 5.7 | 3.8 | 1.9 | 11.0 | 4.1 |
| 3jw8_h1 | 0.4 | 4.5 | 0.6 | 0.7 | 5.7 | 3.2 |
| 3tij_h1 | 6.3 | 9.2 | 2.0 | 5.0 | 8.4 | 2.8 |
| 4hhr_h1 | 1.5 | 4.3 | 5.6 | 3.0 | 9.7 | 9.9 |
| 4hhr_h2 | 0.4 | 0.9 | 6.2 | 2.9 | 4.4 | 6.1 |
| 4hhr_h3 | 2.8 | 3.4 | 8.7 | 6.3 | 5.3 | 8.7 |
| 4m5e_h1 | 1.6 | 3.6 | 0.6 | 1.8 | 3.5 | 0.6 |
| 4nwz_h1 | 3.2 | 0.2 | 11.2 | 8.9 | 0.3 | 11.6 |
| 4qnd_h1 | 4.7 | 3.9 | 1.8 | 4.1 | 3.5 | 3.4 |
| 4rp9_h3 | 3.8 | 6.1 | 1.5 | 3.7 | 7.9 | 6.3 |
| 4umw_h1 | 1.7 | 4.0 | 4.3 | 1.7 | 7.0 | 5.0 |
| 4ymk_h1 | 4.2 | 2.1 | 4.6 | 6.7 | 3.0 | 6.0 |
| 4ymk_h2 | 5.4 | 3.8 | 2.1 | 4.4 | 3.4 | 1.6 |
| 4ymk_h3 | 5.0 | 4.9 | 8.4 | 4.5 | 5.0 | 7.7 |
| 4zwn_h1 | 0.9 | 2.8 | 9.6 | 1.7 | 7.4 | 11.8 |
| 5ahv_h1 | 4.7 | 2.4 | 4.1 | 3.5 | 2.7 | 2.5 |
| 5dqq_h1 | 7.2 | 7.1 | 3.2 | 6.8 | 6.9 | 8.3 |
| 5ek8_h1 | 0.3 | 1.7 | 9.0 | 0.7 | 3.1 | 11.0 |
| 5f19_h3 | 1.9 | 6.9 | 1.9 | 1.8 | 7.0 | 1.8 |
| 5f19_h4 | 0.6 | 9.2 | 15.8 | 6.3 | 9.5 | 16.0 |
| 5lil_h1 | 3.5 | 3.2 | 7.5 | 6.8 | 2.4 | 13.2 |
| 5mlz_h2 | 2.3 | 2.3 | 8.6 | 2.2 | 2.4 | 8.2 |
| 5uz7_h1 | 2.0 | 2.0 | 3.9 | 1.8 | 2.0 | 6.3 |
| 5w7b_h1 | 0.5 | 18.4 | 9.7 | 7.3 | 9.1 | 13.1 |
| 5w7l_h1 | 0.9 | 6.9 | 1.6 | 0.8 | 7.6 | 4.8 |
| 5w7l_h2 | 2.1 | 3.4 | 2.2 | 2.0 | 3.4 | 2.6 |
| 5w7l_h3 | 5.5 | 3.1 | 5.4 | 5.5 | 10.5 | 8.4 |
| 6an7_h1 | 5.3 | 5.9 | 7.9 | 8.4 | 6.4 | 8.7 |
| 6d26_h1 | 0.4 | 1.6 | 4.1 | 1.2 | 1.5 | 5.6 |
| 6dvy_h1 | 2.9 | 3.3 | 9.7 | 2.0 | 3.7 | 12.0 |
| 6igk_h1 | 2.1 | 9.5 | 3.1 | 2.0 | 2.7 | 3.1 |
| Average | 2.9 | 4.7 | 5.5 | 3.6 | 5.2 | 7.0 |
