# Supplementary material for: Prediction of amphipathic helix—membrane interactions with Rosetta
Source: PLoS Comput Biol. 2021 Mar 17;17(3):e1008818. doi: 10.1371/journal.pcbi.1008818 (PMC8007005; doi:10.1371/journal.pcbi.1008818)
Supplement: S6 Table — (DOCX) [file pcbi.1008818.s006.docx]

Supporting Table 6: The MCC values calculated for the best Rosetta zα- and zαβ-scan poses with OPM membrane thicknesses using *RosettaMembrane*, *ref2015*_memb, and *franklin2019* score functions.

| Name | RosettaMembrane MCC zα | ref2015_memb MCC zα | franklin2019 MCC zα | RosettaMembrane MCC zαβ | ref2015_memb MCC zαβ | franklin2019 MCC zαβ |
| --- | --- | --- | --- | --- | --- | --- |
| 1b4v_h1 | 0.00 | 0.00 | 0.00 | 0.00 | 0.00 | 0.00 |
| 1h0a_h1 | 1.00 | 0.45 | 1.00 | 0.27 | 0.45 | 1.00 |
| 1q4g_h1 | 0.69 | 0.69 | 0.83 | 0.69 | 0.69 | 0.83 |
| 1q4g_h2 | 0.39 | 0.39 | 0.19 | 0.26 | 0.54 | 0.19 |
| 1q4g_h3 | 0.36 | 0.15 | 0.43 | 0.29 | 0.15 | 0.43 |
| 1q4g_h4 | -0.38 | -0.39 | 0.12 | 0.63 | 0.12 | 0.12 |
| 1rhz_h1 | 0.59 | 0.58 | 0.54 | 0.59 | 0.58 | 0.33 |
| 2hih_h1 | 0.83 | 0.55 | 0.31 | 1.00 | 0.55 | 0.36 |
| 2ziy_h1 | 0.67 | 0.67 | 0.67 | 0.67 | 0.67 | 0.67 |
| 3a7k_h1 | 0.36 | 0.76 | 0.22 | 0.22 | 1.00 | 0.22 |
| 3hyw_h1 | 0.38 | -0.10 | 0.10 | -0.15 | -0.10 | 0.10 |
| 3hyw_h2 | 1.00 | 0.00 | 0.44 | 0.82 | 0.00 | 0.37 |
| 3i9v_h1 | 1.00 | 0.62 | -0.10 | 0.77 | 0.62 | 0.20 |
| 3j5p_h1 | 0.89 | 0.00 | 0.49 | 0.53 | 0.27 | 0.27 |
| 3jw8_h1 | 1.00 | 0.53 | 0.71 | 0.58 | 0.53 | 0.58 |
| 3tij_h1 | 0.14 | 0.00 | 0.14 | 0.17 | 0.00 | 0.35 |
| 4hhr_h1 | 0.78 | 0.08 | 0.53 | 0.08 | 0.53 | 0.53 |
| 4hhr_h2 | 0.46 | 0.33 | 0.15 | 0.51 | 0.54 | 0.15 |
| 4hhr_h3 | 0.14 | 0.38 | 0.29 | 0.55 | -0.05 | 0.11 |
| 4m5e_h1 | 0.82 | 0.03 | 1.00 | 0.82 | 0.03 | 1.00 |
| 4nwz_h1 | 0.40 | 1.00 | 0.05 | 0.19 | 1.00 | 0.05 |
| 4qnd_h1 | 0.37 | 0.37 | 0.43 | 0.44 | 0.37 | 0.43 |
| 4rp9_h3 | 0.41 | 0.00 | 0.67 | 0.41 | 0.00 | 0.67 |
| 4umw_h1 | 0.63 | 0.43 | 0.43 | 0.63 | 0.43 | 0.43 |
| 4ymk_h1 | 0.15 | 0.28 | 0.53 | 0.53 | 0.53 | 0.53 |
| 4ymk_h2 | 0.00 | 0.65 | 0.15 | -0.15 | 0.65 | 0.00 |
| 4ymk_h3 | 0.49 | 0.52 | 0.52 | 0.56 | 0.52 | 0.52 |
| 4zwn_h1 | 0.41 | 0.55 | 0.09 | 0.34 | -0.09 | 0.09 |
| 5ahv_h1 | 0.37 | 0.45 | 0.45 | 0.29 | 0.45 | 0.45 |
| 5dqq_h1 | 0.17 | 0.17 | 1.00 | 0.17 | 0.17 | 1.00 |
| 5ek8_h1 | 1.00 | 0.59 | 0.11 | 0.80 | 0.40 | 0.11 |
| 5f19_h3 | 0.10 | 0.00 | 0.50 | 0.10 | 0.00 | 0.50 |
| 5f19_h4 | 1.00 | 0.00 | 0.09 | 0.44 | 0.23 | 0.09 |
| 5lil_h1 | 0.27 | 0.34 | 0.11 | -0.24 | 0.39 | 0.13 |
| 5mlz_h2 | 0.63 | 0.63 | 0.17 | 0.63 | 0.47 | 0.12 |
| 5uz7_h1 | 0.67 | -0.06 | 0.00 | 0.59 | -0.06 | 0.00 |
| 5w7b_h1 | 1.00 | 0.14 | 0.35 | 0.50 | 0.14 | 0.14 |
| 5w7l_h1 | 1.00 | 0.22 | 0.80 | 1.00 | 0.22 | 0.80 |
| 5w7l_h2 | 0.68 | 0.36 | 0.37 | 0.84 | 0.68 | 0.60 |
| 5w7l_h3 | 0.33 | 0.53 | 0.53 | 0.33 | 0.53 | 0.53 |
| 6an7_h1 | -0.36 | -0.14 | 0.20 | -0.25 | -0.31 | 0.14 |
| 6d26_h1 | 1.00 | 0.75 | 0.59 | 0.86 | 0.75 | 0.52 |
| 6dvy_h1 | -0.05 | -0.08 | 0.05 | 0.35 | -0.05 | 0.05 |
| 6igk_h1 | 0.82 | 0.17 | 0.49 | 0.56 | 0.82 | 0.82 |
| Average | 0.51 | 0.31 | 0.38 | 0.44 | 0.35 | 0.35 |
