# Supplementary material for: Prediction of amphipathic helix—membrane interactions with Rosetta
Source: PLoS Comput Biol. 2021 Mar 17;17(3):e1008818. doi: 10.1371/journal.pcbi.1008818 (PMC8007005; doi:10.1371/journal.pcbi.1008818)
Supplement: S7 Table — (DOCX) [file pcbi.1008818.s007.docx]

Supporting Table 7: The depths calculated for the OPM structures and the best zα-scan pose calculated with the *RosettaMembrane*, *ref2015_memb*, and *franklin2019* score functions.

| Name | OPM depth | RosettaMembrane zα depth | ref2015_memb depth | franklin2019 depth |
| --- | --- | --- | --- | --- |
| 1b4v_h1 | -4.1 | -3.0 | 7.5 | 2.8 |
| 1h0a_h1 | -3.9 | -4.1 | -5.6 | -2.8 |
| 1q4g_h1 | 1.7 | -1.8 | -2.9 | 0.7 |
| 1q4g_h2 | -3.0 | -1.1 | -2.2 | 4.2 |
| 1q4g_h3 | 2.4 | -1.3 | -5.1 | 0.7 |
| 1q4g_h4 | -5.1 | 3.0 | -3.0 | 7.0 |
| 1rhz_h1 | -3.4 | -3.2 | -5.1 | 0.4 |
| 2hih_h1 | -3.8 | -3.5 | -2.9 | 1.6 |
| 2ziy_h1 | -5.4 | -2.8 | -2.6 | -3.1 |
| 3a7k_h1 | -1.3 | -1.3 | -1.5 | 2.9 |
| 3hyw_h1 | -5.2 | -2.0 | -4.3 | 2.9 |
| 3hyw_h2 | -2.9 | -2.3 | -6.0 | 1.8 |
| 3i9v_h1 | -1.0 | -1.9 | -2.9 | 3.4 |
| 3j5p_h1 | -0.9 | -0.2 | -6.2 | 2.9 |
| 3jw8_h1 | 2.6 | 3.0 | 6.5 | 2.1 |
| 3tij_h1 | 2.2 | -3.7 | -6.1 | 2.5 |
| 4hhr_h1 | 2.5 | 2.7 | 2.0 | 6.3 |
| 4hhr_h2 | -2.0 | -2.5 | -1.9 | 3.5 |
| 4hhr_h3 | -6.5 | -7.9 | -6.5 | 2.1 |
| 4m5e_h1 | -2.7 | -3.9 | -3.1 | -2.3 |
| 4nwz_h1 | -4.6 | -1.5 | -4.4 | 6.4 |
| 4qnd_h1 | 1.6 | -3.0 | -2.0 | 3.1 |
| 4rp9_h3 | 0.8 | -2.9 | -5.0 | -0.6 |
| 4umw_h1 | 0.1 | 0.6 | 3.0 | 3.8 |
| 4ymk_h1 | 3.1 | 2.4 | 3.0 | 7.6 |
| 4ymk_h2 | 4.0 | 0.8 | 4.0 | 2.1 |
| 4ymk_h3 | 3.0 | -1.7 | 5.4 | 11.0 |
| 4zwn_h1 | -3.5 | -3.0 | -6.2 | 5.6 |
| 5ahv_h1 | -0.7 | -3.5 | -2.8 | -1.8 |
| 5dqq_h1 | 5.1 | -1.7 | -1.8 | 3.2 |
| 5ek8_h1 | -2.6 | -2.8 | -3.8 | 5.8 |
| 5f19_h3 | -2.1 | -2.2 | -8.6 | -0.3 |
| 5f19_h4 | -7.9 | -7.3 | -9.5 | 7.0 |
| 5lil_h1 | -4.5 | -1.0 | -1.6 | 2.9 |
| 5mlz_h2 | -5.0 | -3.2 | -2.8 | 3.2 |
| 5uz7_h1 | -5.3 | -3.6 | -6.5 | -9.1 |
| 5w7b_h1 | -4.8 | -5.3 | 13.8 | 4.7 |
| 5w7l_h1 | -2.0 | -2.8 | 4.6 | -0.5 |
| 5w7l_h2 | -0.7 | -2.8 | -4.0 | 1.4 |
| 5w7l_h3 | 1.7 | -3.7 | 4.6 | 6.9 |
| 6an7_h1 | -2.5 | -3.7 | -5.6 | 4.3 |
| 6d26_h1 | -3.9 | -3.6 | -2.4 | 0.0 |
| 6dvy_h1 | -5.0 | -5.0 | -5.1 | 4.5 |
| 6igk_h1 | -2.2 | -0.3 | 7.0 | 0.6 |
