# Supplementary material for: Prediction of amphipathic helix—membrane interactions with Rosetta
Source: PLoS Comput Biol. 2021 Mar 17;17(3):e1008818. doi: 10.1371/journal.pcbi.1008818 (PMC8007005; doi:10.1371/journal.pcbi.1008818)
Supplement: S8 Table — (DOCX) [file pcbi.1008818.s008.docx]

Supporting Table 8: The RMSD values, MCC values, and rotamer recovery rates for χ1 and χ2 belonging to the zα calculations with side chain repacking calculated with the RosettaMembrane score function.

| Helix name | RMSD (Å) | MCC | Rotamer recovery rate (χ1) | Rotamer recovery rate (χ2) |
| --- | --- | --- | --- | --- |
| 1b4v_h1_renum | 2.1 | 0.00 | 0.70 | 0.50 |
| 1h0a_h1_renum | 0.5 | 1.00 | 0.61 | 0.39 |
| 1q4g_h1_renum | 3.6 | 0.69 | 0.64 | 0.46 |
| 1q4g_h2_renum | 3.1 | -0.04 | 0.91 | 0.55 |
| 1q4g_h3_renum | 4.2 | 0.36 | 0.67 | 0.56 |
| 1q4g_h4_renum | 10.2 | -0.26 | 0.53 | 0.24 |
| 1rhz_h1_renum | 2.8 | 1.00 | 0.55 | 0.14 |
| 2hih_h1_renum | 2.0 | 0.83 | 0.50 | 0.19 |
| 2ziy_h1_renum | 2.8 | 1.00 | 0.67 | 0.44 |
| 3a7k_h1_renum | 1.3 | 0.65 | 0.60 | 0.30 |
| 3hyw_h1_renum | 5.0 | -0.17 | 0.73 | 0.33 |
| 3hyw_h2_renum | 2.3 | 0.82 | 0.62 | 0.31 |
| 3i9v_h1_renum | 1.3 | 1.00 | 0.39 | 0.15 |
| 3j5p_h1_renum | 0.9 | 0.76 | 0.47 | 0.27 |
| 3jw8_h1_renum | 0.4 | 1.00 | 0.69 | 0.39 |
| 3tij_h1_renum | 6.1 | 0.14 | 0.63 | 0.38 |
| 4hhr_h1_renum | 1.5 | 0.78 | 0.73 | 0.47 |
| 4hhr_h2_renum | 1.2 | 0.67 | 0.67 | 0.47 |
| 4hhr_h3_renum | 2.9 | 0.14 | 0.79 | 0.58 |
| 4m5e_h1_renum | 1.9 | 0.82 | 0.73 | 0.36 |
| 4nwz_h1_renum | 3.2 | 0.35 | 0.81 | 0.69 |
| 4qnd_h1_renum | 4.6 | 0.37 | 0.80 | 0.60 |
| 4rp9_h3_renum | 3.8 | 0.27 | 1.00 | 0.50 |
| 4umw_h1_renum | 1.6 | 0.63 | 0.60 | 0.50 |
| 4ymk_h1_renum | 4.2 | 0.15 | 0.75 | 0.67 |
| 4ymk_h2_renum | 5.4 | 0.00 | 1.00 | 0.50 |
| 4ymk_h3_renum | 5.0 | 0.41 | 1.00 | 0.70 |
| 4zwn_h1_renum | 0.8 | 0.65 | 0.53 | 0.16 |
| 5ahv_h1_renum | 2.2 | 0.52 | 0.86 | 0.57 |
| 5dqq_h1_renum | 7.2 | 0.17 | 0.33 | 0.13 |
| 5ek8_h1_renum | 1.3 | 1.00 | 0.71 | 0.38 |
| 5f19_h3_renum | 1.9 | 0.10 | 0.89 | 0.56 |
| 5f19_h4_renum | 0.6 | 1.00 | 0.63 | 0.31 |
| 5lil_h1_renum | 3.5 | 0.34 | 0.80 | 0.33 |
| 5mlz_h2_renum | 2.2 | 0.55 | 0.56 | 0.31 |
| 5uz7_h1_renum | 1.8 | 0.34 | 0.68 | 0.27 |
| 5w7b_h1_renum | 11.8 | -0.45 | 0.74 | 0.47 |
| 5w7l_h1_renum | 1.0 | 0.76 | 0.56 | 0.56 |
| 5w7l_h2_renum | 2.2 | 0.84 | 0.55 | 0.27 |
| 5w7l_h3_renum | 5.3 | 0.39 | 0.92 | 0.69 |
| 6an7_h1_renum | 5.3 | -0.31 | 0.38 | 0.06 |
| 6d26_h1_renum | 0.4 | 1.00 | 0.59 | 0.41 |
| 6dvy_h1_renum | 0.3 | 0.46 | 0.59 | 0.41 |
| 6igk_h1_renum | 2.1 | 0.82 | 0.70 | 0.50 |
| Average | 3.0 | 0.49 | 0.68 | 0.41 |
