# Supplementary material for: Prediction of amphipathic helix—membrane interactions with Rosetta
Source: PLoS Comput Biol. 2021 Mar 17;17(3):e1008818. doi: 10.1371/journal.pcbi.1008818 (PMC8007005; doi:10.1371/journal.pcbi.1008818)
Supplement: S9 Table — The thickness calculations were done on full proteins, and the same thickness value was used for all the helices belonging to the same protein structure. Standard deviations were calculated for the different thicknesses given by different methods for the same protein structure. All units are in Ångstroms (Å). (DOCX) [file pcbi.1008818.s009.docx]

Supporting Table 9: Hydrophobic thicknesses calculated by different methods for each protein. The thickness calculations were done on full proteins, and the same thickness value was used for all the helices belonging to the same protein structure. Standard deviations were calculated for the different thicknesses given by different methods for the same protein structure. All units are in Ångstroms (Å).

| PDB ID | OPM | OREMPRO | PDBTM | Stdev |
| --- | --- | --- | --- | --- |
| 1rhz | 14.6 | 14.5 | 14.5 | 0.1 |
| 2ziy | 16.5 | 17.0 | 15.3 | 0.9 |
| 3a7k | 16.9 | 16.0 | 13.8 | 1.6 |
| 3j5p | 14.2 | 16.6 | 16.5 | 1.4 |
| 4qnd | 16.5 | 12.0 | 16.0 | 2.5 |
| 4rp9 | 14.9 | 10.5 | 13.0 | 2.2 |
| 4umw | 15.5 | 11.5 | 12.8 | 2.0 |
| 4ymk | 13.8 | 12.3 | 11.5 | 1.2 |
| 5dqq | 14.9 | 11.1 | 12.5 | 1.9 |
| 5lil | 15.5 | 19.5 | 18.0 | 2.0 |
| 5mlz | 15.3 | 14.3 | 14.8 | 0.5 |
| 5uz7 | 15.1 | 20.0 | 18.0 | 2.5 |
| 6an7 | 14.9 | 15.5 | 14.3 | 0.6 |
| 6igk | 17.1 | 18.0 | 14.8 | 1.7 |
