# Supplementary figures and images for: Prediction of amphipathic helix—membrane interactions with Rosetta
Source: PLoS Comput Biol. 2021 Mar 17;17(3):e1008818. doi: 10.1371/journal.pcbi.1008818 (PMC8007005; doi:10.1371/journal.pcbi.1008818)

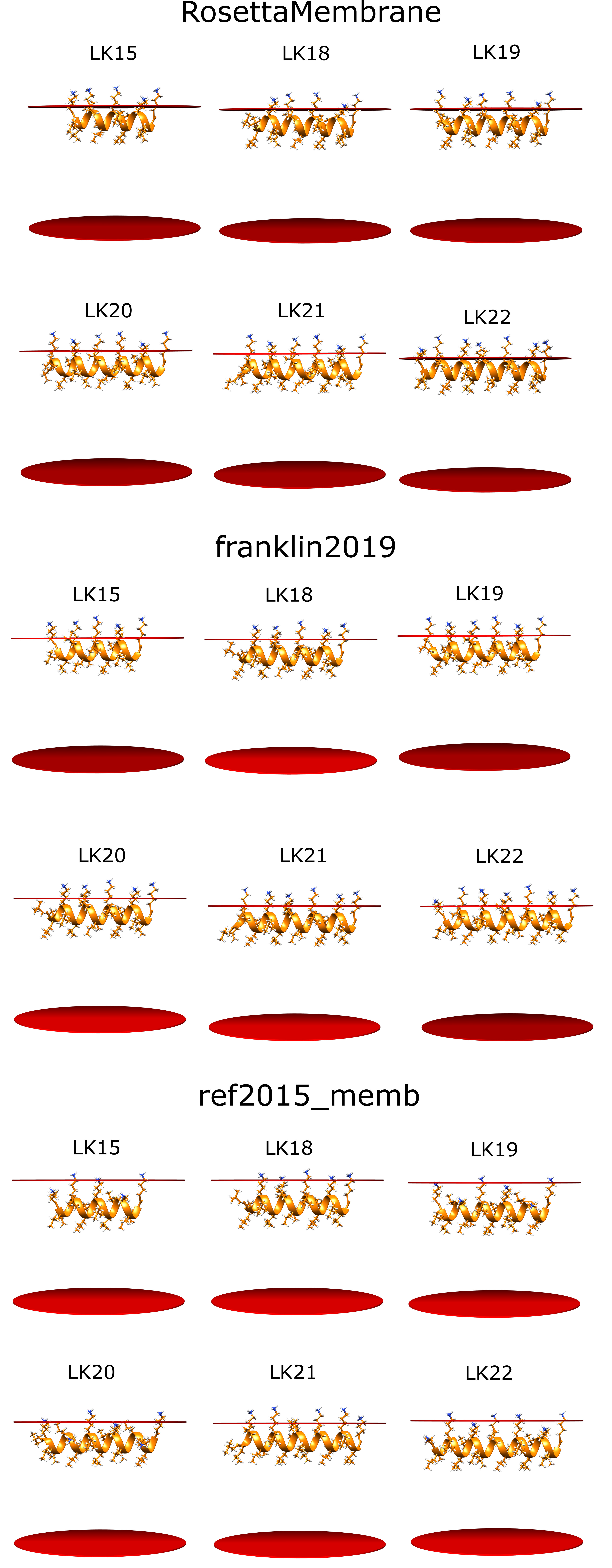

Supplement: S1 Fig — The red planes represent the membrane surface. (TIF) [file pcbi.1008818.s010.tif]

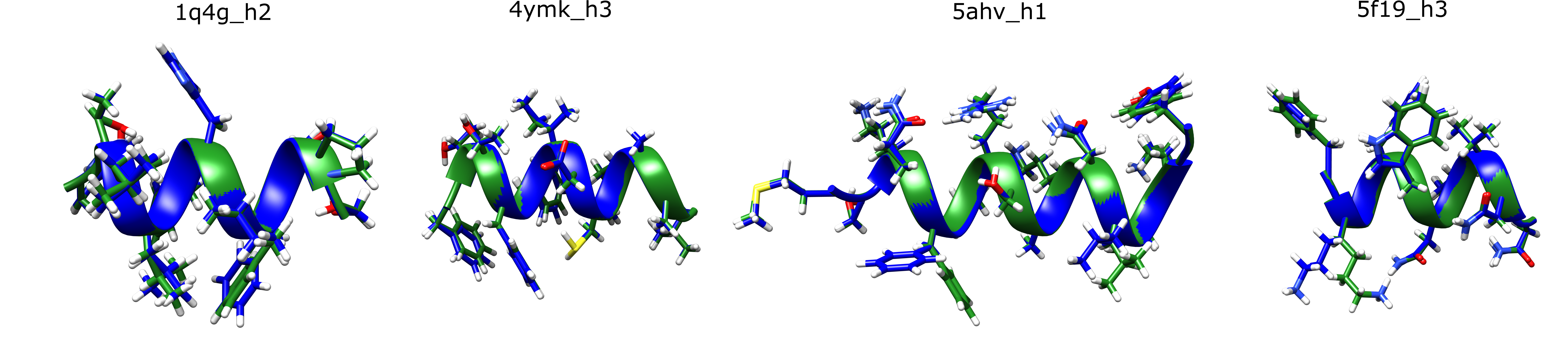

Supplement: S4 Fig — Green color indicates the native structure and blue color indicates the lowest-scoring Rosetta structure. (TIF) [file pcbi.1008818.s013.tif]
